# Novel Elongator Protein 2 Inhibitors Mitigating Tumor Necrosis Factor-*α* Induced Osteogenic Differentiation Inhibition

**DOI:** 10.1155/2021/3664564

**Published:** 2021-11-22

**Authors:** Wen-Jiao Wu, Chang-Liang Xia, Shuan-Ji Ou, Yang Yang, Yun-Fei Ma, Yi-Long Hou, Qing-Po Yang, Jun Zhang, Jian-Wei Li, Yong Qi, Chang-Peng Xu

**Affiliations:** ^1^Department of Medical Research Center, Guangdong Second Provincial General Hospital, Guangzhou, Guangdong, China; ^2^Department of Orthopaedics, Guangdong Second Provincial General Hospital, Guangzhou, Guangdong, China; ^3^Department of Orthopaedics and Traumatology, Nanfang Hospital, Southern Medical University, Guangzhou, Guangdong, China; ^4^Department of Orthopaedics, The First People's Hospital of Kashgar Prefecture, Kashgar, Xinjiang, China; ^5^Department of Orthopaedics, Shenzhen Shekou People's Hospital, Shenzhen, Guangdong, China

## Abstract

Tumor necrosis factor-*α* is a common cytokine that increases in inflammatory processes, slows the differentiation of bone formation, and induces osteodystrophy in the long-term inflammatory microenvironment. Our previous study confirmed that the Elongation protein 2 (ELP2) plays a significant role in osteogenesis and osteogenic differentiation, which is considered a drug discovery target in diseases related to bone formation and differentiation. In this study, we applied an in silico virtual screening method to select molecules that bind to the ELP2 protein from a chemical drug molecule library and obtained 95 candidates. Then, we included 11 candidates by observing the docking patterns and the noncovalent bonds. The binding affinity of the ELP2 protein with the candidate compounds was examined by SPR analysis, and 5 out of 11 compounds performed good binding affinity to the mouse ELP2 protein. After in vitro cell differentiation assay, candidates 2# and 5# were shown to reduce differentiation inhibition after tumor necrosis factor-*α* stimulation, allowing further optimization and development for potential clinical treatment of inflammation-mediated orthopedic diseases.

## 1. Introduction

Persistent bone tissue inflammation, such as bone fractures and rheumatoid arthritis (RA), destroys the bone formation and absorption balance, which reduces bone mass and results in significant impact on bone regeneration [1]. So far, no effective drugs can cope with this clinical problem. We have noticed that elevated tumor necrosis factor-a (TNF-*α*) is a major contributor to the bone pathophysiology through inhibition of osteoblasts function and stimulation of bone resorption activity of osteoclasts [2, 3]. Drug for lessening the potential damage caused by long-term TNF-*α*-associated inflammation exposure is urgent for clinical orthopedics.

Elongation protein 2 (ELP2) is a subordinate of the ELP123 complex and regulates the activity of elongation protein by integrating signals from various factors. It affects the inflammatory response through the JAK-STAT3 cascade and downstream pathways [4]. Our previous research identified the role of ELP2 in regulating TNF-*α*-induced osteoblast differentiation inhibition. A mechanistic study concluded that ELP2 blocked the osteogenic differentiation induced by BMP-2 through activation the STAT3 pathway and downregulating the utterance of BMPR2, thereby slowing the process of early bone tissue formation in the inflammatory microenvironment [5]. Thus, ELP2 was shown to be the potential drug target to develop novel therapeutic for inflammation-induced bone loss.

In silico screening methods, such as quantitative structure-activity relationships (QSAR), pharmacophore models, molecular dynamics (MD) simulations, and high-throughput molecular docking, have been used to find specific protein inhibitors in recent research [6–9]. In our study, we used the molecular docking method to screening novel structure-specific chemicals for ELP2 and obtained eleven chemical compounds, which showed effective binding affinity to ELP2 active pockets. Combined with label-free surface plasmon resonance (SPR) affinity analysis, five candidates showed obvious binding affinity to ELP2. After in vitro experiments, two of the candidate compounds (2 # and 5 #) were shown to block the retardation of osteoblast differentiation induced by TNF-*α*, leading a potent clinical treatment potential for inflammation-induced bone loss.

## 2. Materials and Method

### 2.1. Protein and Ligand Library Preparation

The target protein was obtained from the UniProtKB database (https://www.uniprot.org/) with an entry ID of Q91WG4 (Mus musculus), and the homocrystal structure was selected with the advanced sequence searching function aiming for high similarity. Then, the raw composition of the ELP2 was got from the database (https://www.rcsb.org/) with the identification number 5M2N. The homobuilding step was performed with Structure Prediction Wizard in the Schrodinger software (Maestro, version 2015, Schrödinger LLC, USA). The missing loops and mismatches were corrected using a knowledge-based algorithm provided with the wizard module. To ensure the correct starting structure, the initial structure of the protein 3D model was prepared using the Protein Preparation Wizard module. Hydrogen atoms were increase in structure consistently with physiological pH (7.0). Then, the structure was optimized by adjusting hydrogen bonding and removing atom collisions, and formal charges are added to the heteron and optimized at neutral pH. Finally, the redundant water molecules were removed, and the new structure was minimized using the OPLS-2005 force field optimization potential. The prepared protein structure was subjected to binding site calculation.

The binding sites were generated using the SiteMap tool of the Schrödinger comprehensive assessed according to the calculated attributes, including the size of the site, the degree of protein enclosure, the degree of solvent exposure, the degree of tightness of site interaction with the protein, and the hydrophilic and hydrophobic character of the site. The total site scores were then used as the main basis to select potential binding sites. Finally, we completed the receptor grid generation step ranked the sitemaps from high to low score.

The chemical ligand library, including 552,007 compounds, was retrieved from the InterBioScreen database (InterBioScreen ltd., Russia) in structure data file (.SDF) format (Mar 2018 version). The molecules were arranged to ligand preparation using the LigPrep module with default settings. The ligand preparation process involved saving the definite chiralities and generating five minimum low-energy stereoisomers per ligand with pH values ranging from 5.0 to 9.0.

### 2.2. Virtual Screening

During the docking stage, the ligands were docked to the grids by three-tire-docking [10], which started with HTVS, followed by SP and XP to improve the accuracy. Then, we screened and chose the ligand according to the ranking of the GLIDE score. Herein, the optimized formula of the GLIDE score was as follows: GLIDE score (kcal/mol) = 0.065 × vdW + 0.130 × Coul + Lipo + Hbond + metal + BuryP + RotB + site [11]. The ADME properties of the selected ligands were analyzed using the QikProp tool to conclude the natural properties of the drugs, such as hydrogen bond donor and acceptor even the number of rotatable bonds.

### 2.3. Surface Plasmon Resonance (SPR) Assay

To validate the binding affinity of the candidate compounds with ELP2 protein, we tested the binding affinity through SPR assay. The experiment was performed as previous reported [12]. Briefly, the candidate molecules are crossed onto the chip surface by hydrogen substitution reaction. During the experimental SPR, the chips were first primed with running buffer (1x PBS containing 5% DMSO). Mouse ELP2 protein used in the assay was diluted with running buffer at the concentrations from 200 nM to 3200 nM. To validate the reliability of our method, we selected biotin and rapamycin binding with FKBP12 as a system control. Additionally, the solvents DMSO for compounds and PBS (**pH** = 7.0) for proteins were measured individually as blank controls and background noise controls.

### 2.4. Cell Culture and Treatment

C2C12 cells were kept in DMEM complemented with the 10% fetal dairy serum and 1% penicillin/streptomycin and MC3T3-E1 cells in minimally needed culture medium (MEM, HyClone, GE Healthcare, USA) supplemented with 10% fetal bovine serum and 1% penicillin/streptomycin, were grown individually, and then cultured at 37.0°C, 5% CO_2_. As previously reported [12], cells were cultured for differentiation induction in the presence of TNF-*α* with or without our candidate compounds.

### 2.5. Alkaline Phosphatase Measurement

Bone marrow transplantation was detected by ALP activity via the alkaline phosphatase assay kit followed by the instruction. A BioTek microplate spectrophotometer was used to assay the apparent density at the wavelength of 405 nm. ALP activity values are equally common in the density protein found in stem cells determined to use the BCA Protein Assay Kit.

### 2.6. Mineralization Assessment

As reported, in two differentiation cell models, the culture medium was replaced every 3 days till 35th day. Mineralization of the cell was found by staining using the Optimized Alizarin Red S Stain Kit by the kit instructions.

### 2.7. Real-Time Fluorescent Quantitative PCR

The total mRNA was extracted from cell culture using TRIzol reagent (Thermo Scientific USA). The concentration and quality of the RNA were checked using a NanoDrop™ 8000 spectrophotometer, and cDNA was then synthesized. Gene expression according to the recommendations of the reverse transcription kit was detected. Host gene *β*-actin was selected as the reference gene, and the gene expression level analysis was calculated by the 2^-*ΔΔ*Ct^ quantification method [13].

### 2.8. Western Blot Assay

After treatment, using PBS to wash the cells, the cells were lysed with RIPA lysis buffer. The cell lysate was then separated by SDS-PAGE. All proteins were transferred to PVDF membranes (Bio-Rad, USA). The PVDF membrane was then blocked with 5% skim milk in Tris-buffered physiological saline -0.1% Tween-20 for 1 hour and incubated with the following primary antibody: anti-ALP antibody (rabbit), anti-COL-1 antibody (mouse), anti-OCN antibody (mouse), anti-BMP-2 antibody (mouse), anti-Runx2 antibody (mouse), Anti-Osterix (rabbit), and anti-*β*-actin (mouse), followed by suitable horseradish peroxidase- (HRP-) labeled secondary antibody, blocking buffer for goat anti-rabbit IgG. Images were captured using G&E imaging system.

### 2.9. Statistical Analysis

Multigroup comparison has been made with the two ANOVA methods. A single comparison was made using a one-sided Student's test (*P* < 0.05 was counted as significance). Analysis was completed using SPSS 13.0 Statistics Package and Background 2017. The data is displayed as mean ± SD. The test was tripled and at least once.

## 3. Results

### 3.1. Modelling Preparation and Virtual Screening

**Figure 1:**
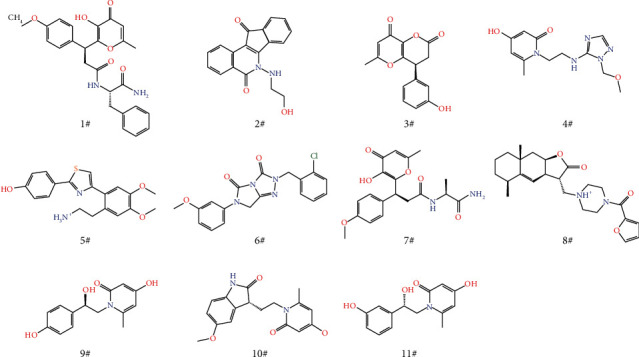
The chemical structures of 11 candidate compounds.

The mouse ELP2 protein structure was homobuilt using PDB entry 5M2N. Missing loops were filled and mismatches were corrected through a knowledge-based algorithm. A preliminary model was then set up using the Protein Preparation Wizard. After further structure optimization, binding sites were generated and receptor grids were calculated by a grid generation module. The chemical ligand library consisting of 552,007 compounds was prepared with the LigPrep module, yielding five minimum low-energy stereoisomers per ligand with pH values ranging from 5.0 to 9.0. In the ligand-docking stage, the ELP2 model and the entire ligand library were first applied to perform a high-throughput virtual screening (HT-VS), and 11,216 compounds were passed. These screened compounds were further analyzed by the standard precision (SP) and extraprecision (XP) glide docking models. 397 hits belonging to 102 clusters were retrieved from the Glide SP docking with a docking score cut-off value of -6.0 kcal*/*mol. Subsequently, the Glide XP module was used to further reduce the hit set, and 95 hits belonging to 36 clusters were retrieved with a docking score cut-off value of -5.0 kcal/mol. Finally, these 95 ligand molecules were visually inspected based on docking poses and bonds interacting with protein residues. Finally, 11 hits were selected as candidate compounds. The structures of the 11 compound candidates are shown in Figure 1. The binding sites and docking surface structures are shown in Supplementary Figure 1A and 1B.

### 3.2. SPR Affinity Analysis

**Figure 2:**
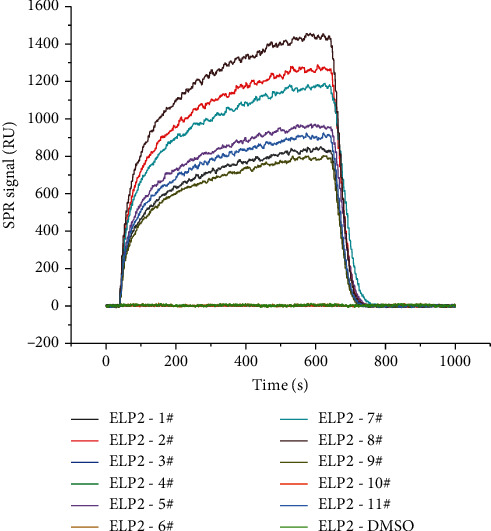
The binding curve of compound 1# to 11# to the mouse ELP2 protein. The selected compounds had been fixed onto the chip surface; ELP2 protein solved in PBS were passed over the chip surface. The raw sensor gram information was collected in real time. The binding of each compound to the ELP2 protein during each cycle is represented by the response unit (RU) of surface resonance.

Under the technique of photo-cross-linker biosensor chip, the candidate compounds were immobilized onto the chemically modified chip surface in random orientations and without any attached label or linker. After the compounds were fixed onto the surface of the chip, ELP2 protein solved in PBS were passed over the chip surface. The original sensor gram information was collected in real time. The association rate constants (*K*_on_), dissociation rate constants (*K*_off_), and the equilibrium dissociation constant (KD) of samples are shown in Table 1. The binding curves are shown in Figure 2. According to the affinity measurement results, five among the eleven candidates possessed strong binding affinity to ELP2, and another two candidates (1# and 9#) showed much faster dissociation rates and were excluded due to presumable difficulty in maintaining adequate efficacy in vivo.

### 3.3. 2# and 5# Compounds Show Remarkable Mitigation against TNF-*α*-Induced ALP Activity Inhibition

**Figure 3:**
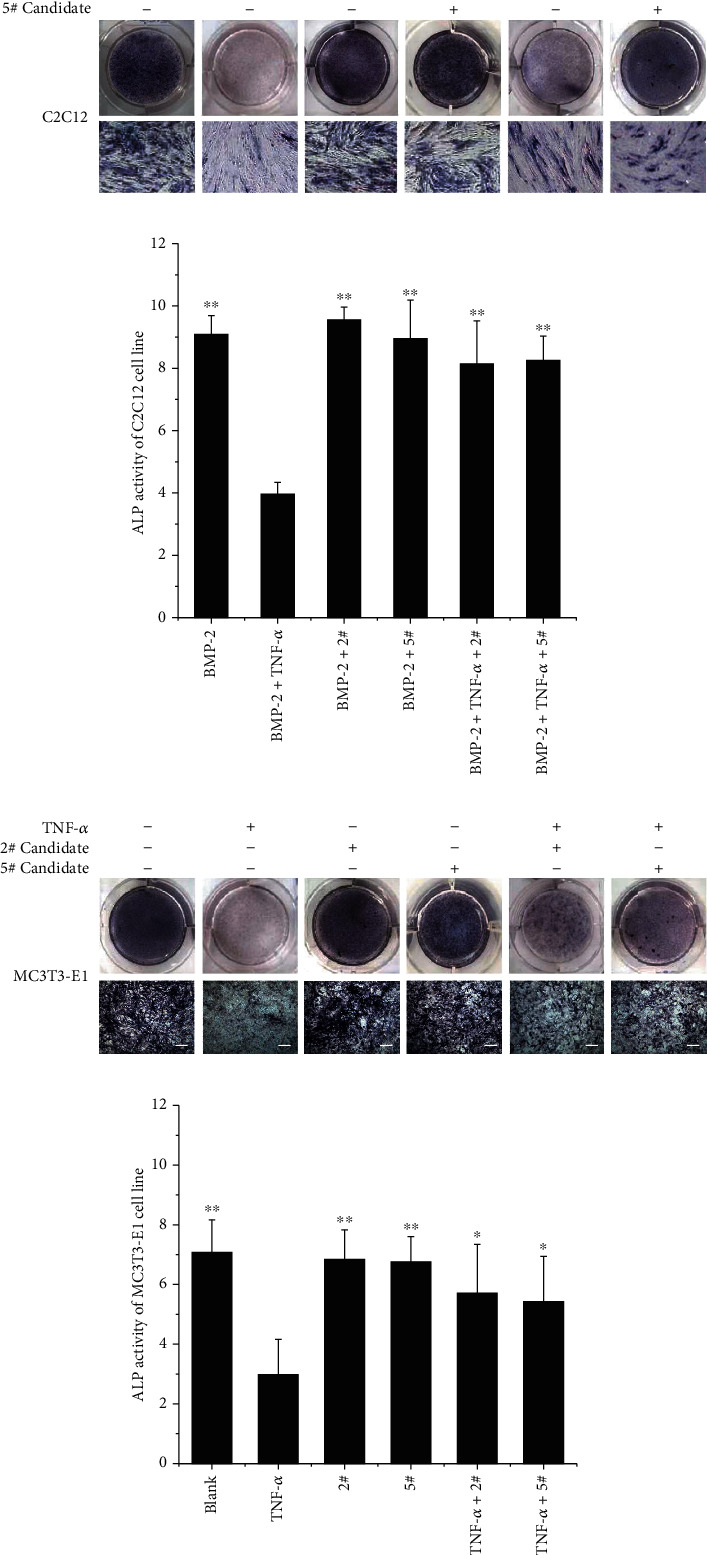
The ALP stain and activity measurements of 2# and 5# candidate on C2C12 and MC3T3-E1 cell osteogenic differentiations. 2# and 5# compounds show remarkable mitigation against TNF-*α* induced ALP activity inhibition. The ALP staining observation in each group (a, c). The relative ALP activity were measured from the cell lysates of each group (b, d). Data are mean ± SD; ^∗^*P* < 0.05, ^∗∗^*P* < 0.01, each experiment group vs. the TNF-*α* treatment cell group.

**Figure 4:**
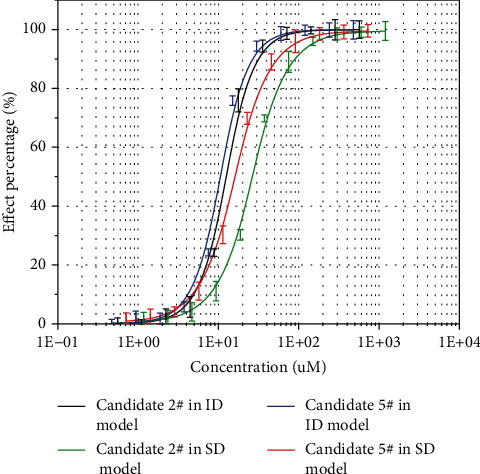
The EC_50_ values of candidates 2# and 5# were calculated and drafted as the dose-effect curves.

To determine the effect of the candidate compounds on TNF-*α*-induced osteogenic differentiation inhibition, we examined the activity of osteogenic marker alkaline phosphatase in the cells. As mentioned in Materials and Method, cells were grown in an adequate osteoblast differentiation environment and tested the alkaline phosphatase activity. Staining observations and relative ALP activity are showed in Figures 3. The results indicated that compared with the osteogenic differentiation controls of the ID and SD differentiation models, TNF-*α* significantly reduced ALP activity and lightened the color after ALP staining, while the presence of candidates 2# and 5# brought a significant ease to TNF-*α*-induced inhibition of osteoblast differentiation in C2C12 (Figures 3(a) and 3(b)) and MC3T3-E1 cell (Figures 3(c) and 3(d)). The effects of candidates 7#, 8#, and 11# were not as strong as 2# and 5#, with only slightly easing of the inhibition resulting from TNF-*α* (data not shown). The half-effective concentration (EC_50_) of candidates 2# and 5# were measured through examining the ALP activity (Figure 4); the EC_50_ of 2# candidate was 14.71 *μM* in C2C12 cells of the ID model; at the same time, the EC_50_ in the MC3T3-E1 SD model was 29.68 *μM*. The EC_50_ values of 5# candidate were 12.15 *μM* and 18.24 *μM* in ID and SD model cells, respectively. Candidate 5# possessed better protective effect compared with candidate 2#, which was consistent with SPR binding affinity test result that 5# showed higher binding affinity to ELP2 protein (Figure 2).

### 3.4. 2# and 5# Compounds Reverse TNF-*α*-Induced Inhibition on Osteoblast Mineralization Activity

**Figure 5:**
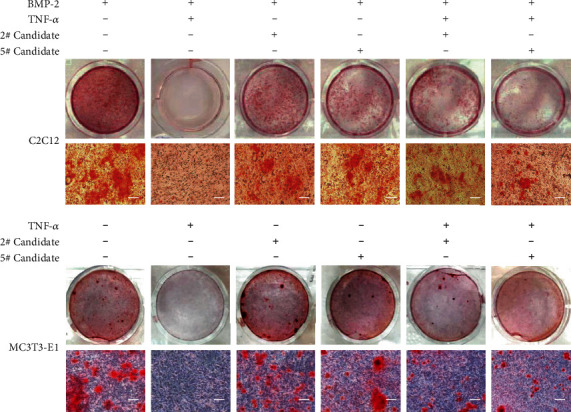
Alizarin Red S staining of differentiated osteogenic cells, the self-differentiation (SD) model, and inflammatory-differentiated (ID) model cells were stained with Alizarin Red S staining on day 35 after differentiation induction, and the mineralization activity of each group can be estimated by the number of stained spots (bar = 50 *μ*m).

In order to test the function of the candidate compounds on the mineralization activity during osteoblasts differentiation, the self-differentiation (SD) model and inflammatory-differentiated (ID) model cells were stained with Alizarin Red S staining on day 35 after differentiation induction, and the mineralization activity of each group can be estimated by the number of stained spots. As Figure 5 has shown, extracellular mineralization was stained with Alizarin Red S staining; compared with the TNF-*α* control groups, candidates 2# and 5# increased the red spot on the cell surface, suggesting that cell mineralization can be reversed by the presence of candidates 2# and 5# in C2C12 and MC3T3-E1 cells (Figure 5), and we inferred that the TNF-*α*-induced osteoblast differentiation inhibition was reversed based on the appearance of mineralization activity changes in the cell models.

### 3.5. 2# and 5# Compounds Increased the Expression Level of Osteoblast Differentiation-Related Marker after TNF-*α* Stimulation

**Figure 6:**
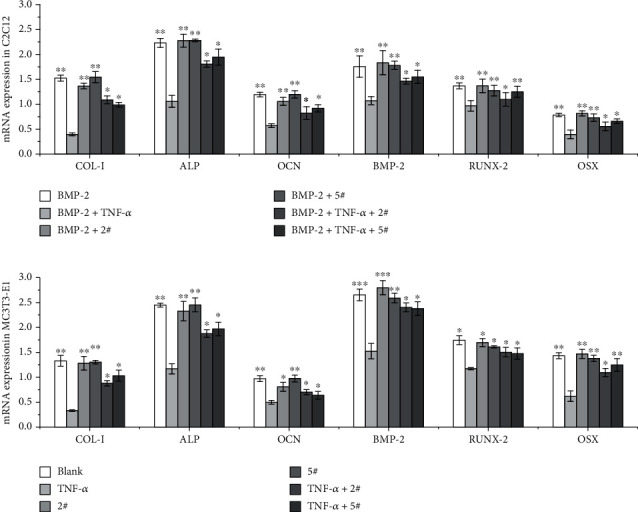
Effect of 2# and 5# treatment on TNF-*α*-stimulated C2C12 (a) and MC3T3-E1 (b) cell osteoblast differentiation-associated marker gene expression. Q-PCR analysis in the differentiation was conducted on the 7th day after osteoblast cell differentiation in the ID and SD models. Data are mean ± SD; ^∗^*P* < 0.05, ^∗∗^*P* < 0.01, each experiment group vs. the TNF-*α* group.

**Figure 7:**
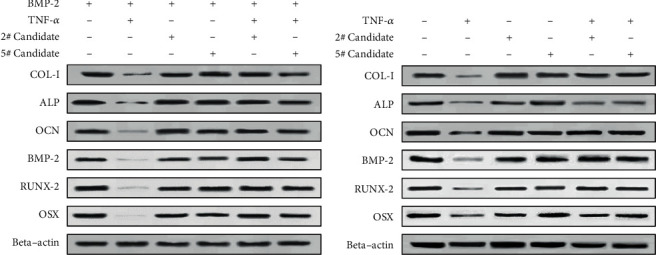
The expression of osteogenic differentiation markers were increased by the 2# and 5# drug candidates compared with TNF-*α* treatment in the cell differentiation process. The protein of the differentiation model cells was harvested on the 7th day after osteoblast cell differentiation and then subjected to Western blot analysis.

In order to investigate the changes in osteogenic differentiation associated molecules including COL-I, ALP, OCN, BMP-2, RUNX-2, and OSX genes, we performed Q-PCR and Western blot analysis on the differentiation cellular models on the 7th day after osteoblast cell differentiation are shown in Table 2. According to the difference of the gene expression levels, the differentiation control group was significantly upregulated compared with TNF-*α* treated cells on the day 7. The level of above gene expression was raised in the 2# and the 5# candidate-treated cell with cell morphology phenotype differentiation when compared to the cell stimulated with TNF-*α* (Figure 6). A further study of the expression of osteoblastic markers at the protein level was performed using Western blot. As shown in Figure 7, the expressions of the marker proteins were consistent with the mRNA expressions. Therefore, our above experiments indicated that the 2# and 5# ELP2 inhibitor candidates could effectively lighten the TNF-*α*-induced inhibition of primary osteoblast differentiation during bone inflammation.

## 4. Discussion

With different bone cells working together, it completes the role of absorbing the old bone and generating a new bone. On this way, the morphology and structure of the bone will be constantly destroyed and remodeled, and this depends on the number and proportion of cells, so the differentiation of osteoblasts can affect the morphology and structure of bone. Persistent inflammatory bone environment hinders osteoblastic differentiation, leading to bone dysplasia [14–16]. TNF-*α* is a main contributor to persistent inflammation- and infection-induced bone regeneration inhibitions; the underline mechanism is still not clear [17]. A previous study [6] reported that ELP2 positively regulated the increased TNF-*α*-induced repression of osteoblast differentiation during persistent inflammation. In clinical studies, the repression of osteoblast differentiation will affect the regeneration and formation of bone. Therefore, ELP2 can inhibit bone dysplasia induced by TNF-*α*, which leads it playing a role in long-term chronic infections.

Molecular docking methods are an effective method to identify potential chemical structures for novel targets [18]. Compared with traditional high-throughput screening, the computational calculation method is faster and more cost-effective. The Schrödinger suit is also an efficient ligand screening program, which is widely used and has been proven in the field of ligand-seeking research [19, 20]. In this study, we performed virtual docking following the published method and using the IBS compound library and obtained 11 candidates that fit into the active site of the homobuilt mouse ELP2 protein. These candidate compounds were prepared for SPR affinity analysis with ELP2 protein, which is a technique that enables rapid detection of the interaction between two molecules together with their binding kinetic parameters in real time [21]. Up to now, no research has reported the epitope of ELP2 active center; therefore, we simulated some possible active centers using the simulation method with the protein structure building module. It is undeniable that such a method results in a large number of incorrect active centers and ineffective ligand candidates in the screening. We speculated that this was the reason only 2 of the 11 high-scored candidate compounds showed inhibition activity in the *in vitro* experiments.

During our experiment, we applied an *in silico* virtual screening to select compound molecules from the chemical drug library that bind to the ELP2 protein and obtained 95 candidates. We further filtered out 11 candidates by scoring the docking model and observing noncovalent bonds. Afterwards, the binding affinities of the ELP2 protein with candidate compounds were analyzed by SPR assay, and 5 of the 11 compounds possessed obvious binding affinity to ELP2 protein. Accordingly, these 5 potential compounds were used for the in vivo efficiency study to determine whether there was an associated potential drug effect. The candidate was tested in the differentiation model and verified, which are used for evaluation of osteoblast differentiation [22]. Further, Q-PCR and Western blot analyses were used to investigate the osteogenic differentiation markers which was affected by the two compounds; the results were consistent with previous, which indicated that 2 compounds could mitigate the effect of TNF-*α*-induced differentiation inhibition.

In summary, we obtained two competitive inhibitors of ELP2 protein; both of them have the potential to impede TNF-*α*-induced new bone-forming osteoblasts (OB) inhibition in two cells. The EC_50_ of candidate 2 # was 14.71 *μ*M in the C2C12 cells of the ID model, while the EC_50_ in the MC3T3-E1 SD model was 29.68 *μ*M. Candidate 5 # EC_50_ values were 12.15 *μ*M and 18.24 *μ*M in the ID and SD model cells, respectively. The results about the bone differentiation assay were in line with expectations. The results give hope that the 2 # and 5 # candidates will become new drugs in clinical orthopedics. Finally, further research will spare no effort to clarify the additional pharmacodynamics and metabolic kinetics of the 2# and 5# candidates, so as to develop them into clinical drugs that prevent the inhibition of bone regeneration caused by persistent inflammation.

## Figures and Tables

**Table 1:** Affinity constants of candidate compounds with ELP2 protein.

No.	Protein	Compound	Avg *K*_on_ (M^−1^ s^−1^)	Avg *K*_off_ (s^−1^)	Avg KD (M)
1#	Elp2	1#	1.98*E* + 03	2.34*E* − 01	1.18*E* − 04
2#	Elp2	2#	1.65*E* + 02	2.41*E* − 04	1.46*E* − 06
3#	Elp2	3#	2.31*E* + 00	4.50*E* − 01	1.95*E* − 01
4#	Elp2	4#	1.85*E* + 00	3.42*E* − 01	1.85*E* − 01
5#	Elp2	5#	3.72*E* + 03	1.22*E* − 02	3.28*E* − 06
6#	Elp2	6#	2.54*E* + 00	4.15*E* − 01	1.63*E* − 01
7#	Elp2	7#	3.47*E* + 02	1.10*E* − 03	3.17*E* − 06
8#	Elp2	8#	1.12*E* + 04	1.05*E* − 03	9.36*E* − 08
9#	Elp2	9#	5.02*E* + 02	7.50*E* − 02	1.49*E* − 04
10#	Elp2	10#	2.18*E* + 00	5.04*E* − 01	2.32*E* − 01
11#	Elp2	11#	2.22*E* + 02	7.74*E* − 04	3.48*E* − 06
Blank	Elp2	DMSO	1.39*E* + 00	9.03*E* − 01	6.51*E* − 01

**Table 2:** Primer sequences of osteogenic differentiation marker genes.

Gene symbol	Primer sequence (5′-3′)	Product size (bp)	Accession no.
COL-I	F: 5′-GACCCTAACCAAGGATGCAA-3′	200	NG_007404.1
	R: 5′-GGAAGTTCAGGATTGCCGTA-3′		
ALP	F: 5′-CCACGTCTTCACATTTGGTG-3′	196	NG_008940.1
	R: 5′-AGACTGCGCCTGGTAGTTGT-3′		
OCN	F: 5′-GTGCAGAGTCCAGCAAAGGT-3′	202	NG_047015.1
	R: 5′-CGATAGGCCTCCTGAAAGC-3′		
BMP-2	F: 5′-TCAAGCCAAACACAAACAGC-3′	197	NG_023233.1
	R: 5′-ACGTCTGAACAATGGCATGA-3′		
RUNX-2	F: 5′-CTCTTCCCAAAGCCAGAGTG-3′	206	NG_008020.1
	R: 5′-CAGCGTCAACACCATCATTC-3′		
OSX	F: 5′-TAATGGGCTCCTTTCACCTG-3′	198	NG_023391.2
	R: 5′-GAGCCATAGGGGTGTGTCAT-3′		

## Data Availability

Once upon request, the corresponding author will offer the data included in this study.

## Abbreviations

COL-I: Collagen alpha-1
EC_50_: Effective concentration 50
EC: Elongator complex
ELP123: Elongator subunits 1-3
ELP456: Elongator subunits 4-6
ELP2: Elongator protein 2
FBS: Fetal bovine serum
GLIDE: Grid-based ligand docking with energetics
HRP: Horseradish peroxidase
HTVS: High-throughput virtual screening
ID model: Inflammatory differentiation model
KD: Dissociation constant, *K*_on_/*K*_off_
MD: Molecular dynamics
OCN: Osteocalcin
OSX: Osterix
Pol II: RNA polymerase II
QSAR: Quantitative structure-activity relationships
RA: Rheumatoid arthritis
RU: Response units
RUNX-2: Runt-related transcription factor 2
SD model: Self-differentiation model
SLE: Systemic lupus erythaematosus
SP: Standard precision
SPR: Surface plasmon resonance
StIP1: STAT3-interacting protein 1
XP: Extraprecision mode.

## Supplementary Materials

Supplementary Materials Supplementary Figure 1: the binding sites and docking surface structures of eleven candidates in docking procedure.
