# Supplementary material for: Novel Elongator Protein 2 Inhibitors Mitigating Tumor Necrosis Factor-α Induced Osteogenic Differentiation Inhibition
Source: Biomed Res Int. 2021 Nov 22;2021:3664564. doi: 10.1155/2021/3664564 (PMC8629650; doi:10.1155/2021/3664564)
Supplement: Supplementary Materials — Supplementary Figure 1: the binding sites and docking surface structures of eleven candidates in docking procedure. [file 3664564.f1.docx]

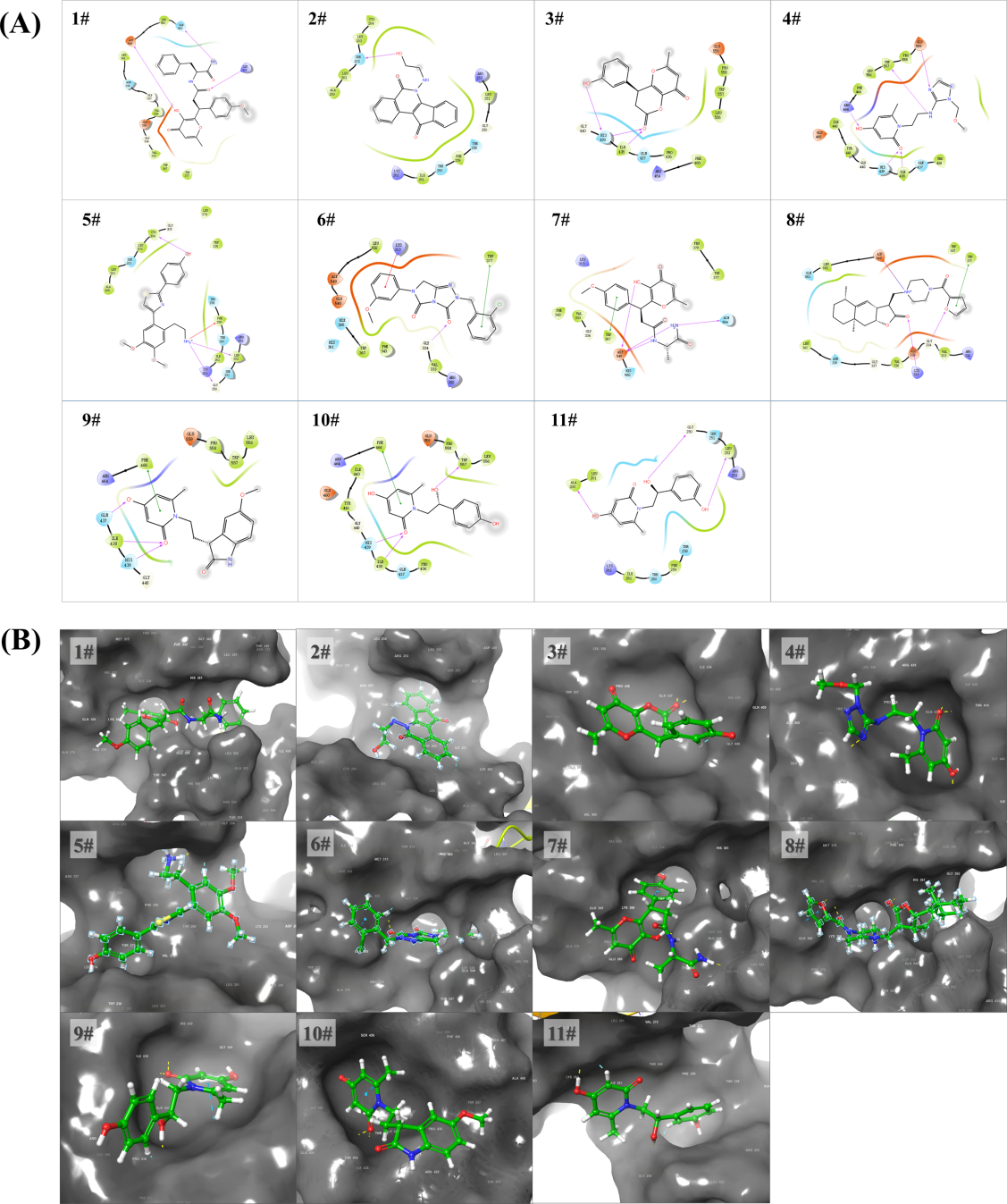


Supplementary Figure 1. The binding sites (A) and docking surface structures (B) of each compound to the ELP2 protein.
